# Local synthesis of interferon-alpha in lupus nephritis is associated with type I interferons signature and LMP7 induction in renal tubular epithelial cells

**DOI:** 10.1186/s13075-015-0588-3

**Published:** 2015-03-22

**Authors:** Giuseppe Castellano, Cesira Cafiero, Chiara Divella, Fabio Sallustio, Margherita Gigante, Paola Pontrelli, Giuseppe De Palma, Michele Rossini, Giuseppe Grandaliano, Loreto Gesualdo

**Affiliations:** Renal, Dialysis and Transplantation Unit, Department of Emergency and Organ Transplantation, University of Bari, Piazza Giulio Cesare 11, 70124 Bari, Italy; C.A.R.S.O. Consortium, Valenzano, Bari Italy; Renal, Dialysis and Transplantation Unit, Department of Medical and Surgical Sciences, University of Foggia, Foggia, Italy

## Abstract

**Introduction:**

Type I interferons are pivotal in the activation of autoimmune response in systemic lupus erythematous. However, the pathogenic role of interferon-alpha in patients affected by lupus nephritis remains uncertain. The aim of our study was to investigate the presence of a specific interferon signature in lupus nephritis and the effects of interferon-alpha at renal level.

**Methods:**

We performed immunohistochemical analysis for MXA-protein and *in situ* hybridization to detect interferon-alpha signature and production in human lupus nephritis. Through microarray studies, we analyzed the gene expression profile of renal tubular epithelial cells, stimulated with interferon-alpha. We validated microarray results through real-time polymerase chain reaction, flow cytometry on renal tubular epithelial cells, and through immunohistochemical analysis and confocal microscopy on renal biopsies.

**Results:**

Type I interferons signature was characterized by MXA-specific staining in renal tubular epithelial cells; in addition, *in situ* hybridization showed that renal tubular epithelial cells were the major producers of interferon-alpha, indicating a potential autocrine effect. Whole-genome expression profile showed interferon-alpha induced up-regulation of genes involved in innate immunity, protein ubiquitination and switching to immunoproteasome. In accordance with the *in vitro* data, class IV lupus nephritis showed up-regulation of the immunoproteasome subunit LMP7 in tubular epithelial cells associated with type I interferon signature.

**Conclusions:**

Our data indicate that type I interferons might have a pathogenic role in lupus nephritis characterized by an autocrine effect of interferon-alpha on renal tubular epithelial cells. Therefore we hypothesize that inhibition of type I interferons might represent a therapeutic target to prevent tubulo-interstitial damage in patients with lupus nephritis.

**Electronic supplementary material:**

The online version of this article (doi:10.1186/s13075-015-0588-3) contains supplementary material, which is available to authorized users.

## Introduction

Systemic lupus erythematous (SLE) is an autoimmune disease characterized by the involvement of several organs including kidneys, joints, nervous system, heart and lung [1,2]. Lupus nephritis remains a common complication and a major determinant of the outcome in SLE patients [3]. The pathological manifestations of lupus nephritis are diverse and involve different renal compartments, including glomeruli, tubules, interstitium and vessels [3,4]. Next to glomerular injury, tubular damage has a significant impact on prognosis and renal function [5].

Hyperactivation of dendritic cells (DC), in particular of the plasmacytoid DC subset, plays a pivotal role in the autoimmune response of SLE [6-9]. The serum of SLE patients contains immune complexes with DNA or RNA molecules that can induce the activation of plasmacytoid DC, resulting in an increased production of interferon-alpha (IFN-alpha) and migration to inflamed tissues [10]. Interferons are ubiquitous cytokines and comprise three major classes: type I (alpha and beta), type II (gamma) and type III (lambda), each type with its specific receptor and signal transduction pathways [11]. Interferons have a central role in the pathogenesis of several autoimmune diseases, such as rheumatoid arthritis, multiple sclerosis and diabetes [7].

Recently, we have demonstrated a tubulo-interstitial plasmacytoid DC infiltrate in the kidney of patients with lupus nephritis [12,13]. Tubulo-interstitial injury is considered to be an important predictor of renal damage in lupus nephritis [4]. In this context, infiltrating leukocytes might release inflammatory molecules that, in turn, regulate nuclear factor-kB (NF-kB) activation, thus contributing to the progression of tissue injury [3,5].

Despite the increasing evidence of a central role for type I IFN in the pathogenesis of SLE, the role of IFN-alpha in the development of lupus nephritis remains uncertain [3,11]. Herein, we investigate the possible pathogenic role of IFN-alpha in SLE patients affected by lupus nephritis.

## Methods

### Immunohistochemistry

Renal tissue samples were obtained from sixteen SLE patients with lupus nephritis who underwent kidney biopsy in the period 2005 to 2012 (eight classes I–II, eight class IV). The study was approved by the Ethical Committee of Azienda Ospedaliera Universitaria Consorziale Policlinico di Bari (study number: RF-1470765) and informed consent was obtained from patients according to the Declaration of Helsinki.

Paraffin-embedded sections of biopsies were deparaffinized and underwent epitope unmasking by pressure-cooking in citrate buffer (0.01 M, pH = 6). Then the slides were incubated with H_2_O_2_ (3%), triton (0.05%), protein block solution (Dako Cytomation, Glostrup, Denmark) and with the primary anti-LMP7 (1:500, Enzo Life Sciences, Inc., Farmingdale, NY, USA) and anti-MXA (1:200, Abcam, Cambridge, UK) antibodies. The binding of the secondary biotinylated antibodies was detected by the Dako Real EnVision Detection System, Peroxidase/DAB kit (Dako Cytomation), according to the manufacturer’s instructions. Visualization of peroxidase was achieved by incubation in 3,3′-diaminobenzidine (DAB) Chromogen Solution, producing a brown precipitate. The sections were counterstained with Mayer hematoxylin (blue) and mounted with glycerol (Dako Cytomation). Negative controls were obtained by substituting the primary antibody with a control irrelevant immunoglobulin G (IgG). Digital images from the experimental glass slides were obtained using ScanScope Digital Slide Scanner (Aperio, Vista, CA, USA) at a 20× magnification and archived on the devoted Spectrum Server V10.2.2.2315 (Aperio). Quality control of the scanned images and all further analyses were performed using ImageScope V10.2.1.2315 (Aperio). Specific tubular LMP7 and MXA immunostaining was quantified using Adobe Photoshop software and expressed as % marked area/total area.

### Hybridization *in situ*

Hybridization *in situ* (ISH) mRNA detection was performed on 4 μm-thick seriate sections from paraffin-embedded human renal biopsies. Slides from SLE patients with lupus nephritis (four classes I–II, four classes IV) were deparaffinized in xylene and ethanol solutions at room temperature and then fixed in 4% paraformaldehyde for 15 minutes in the dark. The permeabilization step was performed by incubating the slides with Proteinase K (15 μg/ml) for 10 minutes at 37°C. Slides were dehydrated in new ethanol solutions and washed in PBS pH 7.4 after each step.IFN-alpha digoxigenin-labeled LNA™-enhanced detection probe (Probe_IFNA_1/5DigN/TTTGCTTTCCTTCATGCACTCT/3Dig_N/ -custom LNA™, Exiqon, Vedbaek, Denmark) was denatured for 75 seconds at 80°C, then the hybridization step was performed adding the probe diluted 40 nM in the hybridization mix (ISH Buffer, Mercury LNA micro RNA ISH, Exiqon) in a humidified chamber for one hour at 50°C. Hybridized slides were then washed with Saline-sodium citrate buffer (*SSC*) at 55°C. Positive and negative controls were obtained by incubating serial sections with 5′-DIG labeled Scramble-ISH and 5′-DIG labeled ß-actin LNA™ mRNA *in situ* hybridization probes, respectively (5 moll, Exiqon). Signal amplification was obtained with a specific kit (Mercury LNA micro RNA ISH, Exiqon) following the manufacturer’s instructions. Hybridized slides were blocked with blocking buffer for 30 minutes at room temperature and then treated with sheep anti-DIG-AP (anti-Digoxigenin-AP, Fab fragments, Roche Diagnostic GmbH, Mannheim, Germany) diluted 1:800 in dilution buffer for one hour at room temperature. Finally, slides were incubated with AP substrate reagent (NBT/BCIP tablets, Roche Diagnostic GmbH) for two hours at 30°C and then the reaction was stopped with KTBT buffer. The sections were counterstained with Nuclear Fast Red and mounted with glycerol (Dako Cytomation). Digital images from the experimental glass slides were obtained using ScanScope Digital Slide Scanner at a 20× magnification and archived on the devoted Spectrum Server V10.2.2.2315.

### Confocal laser scanning microscopy

Paraffin-embedded human kidney sections and primary human renal proximal tubular cells (RPTECs) were stained or double stained for LMP7, MXA, p65, pNIK (Santa Cruz Biotechnologies, Santa Cruz, CA, USA) and BDCA2 (Miltenyi Biotec, Calderara di Reno, Italy). For each experiment, 3 × 10^5^ cells were plated on a cover slip and fixed in 3.7% paraformaldehyde. The slides were incubated with the blocking solution, primary antibodies (anti-LMP7 1:500; anti-MXA 1:200; anti-p65 1:100, anti-pNIK 1:100 and anti -BDCA2 1:20) and the appropriate secondary antibodies (Alexa Flour 488 and 555 goat anti rabbit; Alexa Flour 555 and 488 anti mouse, Molecular Probes, Eugene, OR, USA). All sections were counterstained with TO-PRO-3 (Molecular Probes). Specific fluorescence was acquired using the confocal microscope Leica TCS SP2 (Leica, Wetzlar, Germany) using a 63 objective lens.

Quantification of p65 and pNIK nuclear fluorescence intensity was performed using Leica Software. We outlined cellular nuclei and then calculated the fluorescence intensity for p65 (green channel) and pNIK (red channel) within the nuclei. At least 10 cells in three different fields from each slide were measured to obtain the average quantification of nuclear signal intensity for p65 and pNIK.

### Cell culture

RPTECs were purchased from Lonza (Lonza Group Ltd, Basel, Switzerland) and maintained in the recommended medium, REGM (Lonza) containing renal epithelial cell basal medium supplemented with human epidermal growth factor (hEGF) 0.1%; hydrocortisone 0.1%; epinephrine 0.1%; insulin 0.1%; triiodothyronine 0.1%; transferrin 0.1%; gentamycin/amphotericin-1000 0.1% and fetal bovine serum (FBS) 0.5%. Cells were used between passages 4 and 7 and were plated at a density of 350,000 cells/well in six-well plates (Corning Life Sciences, Acton, MA, USA). Human recombinant IFN alpha (I4676-20 UG Sigma-Aldrich, Milan, Italy) was applied to the RPTEC at 100 U/ml for 48 hours in microarray analysis and for 24 hours and 48 hours in real time polymerase chain reaction (RT-PCR) and western blot at 100 U/ml for 48 hours in flow cytometry analysis and for time-course in immunocytochemistry analysis. RPTEC were processed for p65 and pNIK protein expression by immunofluorescence and confocal analysis and for LMP7 and HLA protein expression by flow cytometry analysis. In different sets of experiments RPTEC were lysed for RNA and protein extraction.

### Microarray analysis

Microarray analysis was performed as previously described [14]. The Illumina microarray data are MIAME (Minimum Information About a Microarray Experiment) compliant and the raw data are available under accession number GSE48551 at the Gene Expression Omnibus (GEO). Raw data were imported into the Genome Studio Data Analysis Software and quality controls were performed. Genes displaying differential expression between IFN-alpha treated cells and untreated cells were detected by the false discovery rate (FDR) method of Storey and Tibshirani [15]; gene probe sets were sorted on the basis of the FDR, (adjusted-*P* value with multiple testing on 1,000 permutations) and fold-change. Only genes that were significantly (adjusted-*P* value <0.05 and fold-change >2) modulated were considered for further analysis. The Gene Set Enrichment Analysis (GSEA) data-mining technique was used to determine whether there was coordinated differential expression or ‘enrichment’ in a set of functionally related genes when comparing control and experimental samples [16]. In the current study, sets of *a priori*, user-defined functionally related genes were included in the GSEA as well as normalized gene expression data from IFN-alpha treated RPTEC versus control analyses. C2 curated gene sets from the Broad Institute, based on prior biological knowledge, sharing a common function, were used for the analysis. Significance of differential expression, as determined by the enrichment analysis, was recalculated 1,000 times. A corrected *P*-value was obtained from the analysis using the FDR q-value correction. On the basis of this correction, the cutoff for significance was established at a *P*-value <0.05. Microarray analysis was performed using Gene Spring GX 11.0 (Agilent Technologies Inc., Santa Clara CA, US).

### RNA extraction and real time PCR analysis

Total RNA was isolated using the RNeasy Mini Kit (Qiagen, Hilden, Germany) according to the manufacturer’s instructions and quantified by NanoDrop ND-1000 Spectrophotometer (NanoDrop Technologies, Inc., Wilmington, DE, USA). Its quality was assessed by electrophoresis on agarose gel (1%). Then, 0.5 μg of total RNA was used in a reverse transcription reaction using the Quantitect reverse transcription Kit (Qiagen) according to the manufacturer’s instructions. Quantitative RT-PCR was performed on an iCycler Thermal Cycler (Bio-Rad Laboratories, Hercules, CA, USA) using the Hs_PSMB8_1_SG, Hs_DTX3L_1_SG and Hs_FBXO6_1_SG QuantiTect Primer Assay (QIAGEN) in combination with SYBR Green dye. The relative amounts of PSMB8, DTX3L and FBXO6 mRNA were normalized to β-actin mRNA as housekeeping gene.

### Immunophenotypic analysis

To analyze surface expression of HLA-I Ag, cells were washed and resuspended in FACS buffer (phosphate- buffered saline pH 7.2, 0.2% bovine serum albumin, and 0.02% sodium azide), then incubated with phycoerythrin (PE)-conjugated-HLA-I (Beckman Coulter) for 15 minutes at 4°C, and finally washed with the same buffer before flow cytometry analysis.

For intracellular staining of LMP7, unconjugated monoclonal antibody (Enzo Life Sciences) was used. Intracellular staining was preceded by fixation and permeabilized with Intraprep reagents (Beckman Coulter) and incubated for 25 minutes at 4°C with the primary antibody. Cells were then washed and incubated with the secondary antibody Alexa Fluor 488 (Molecular Probe) for 25 minutes.

Data were acquired using a FC500 (Beckmann Coulter) flow cytometer and analyzed using CXP software. The area of positivity was determined using an isotype-matched monoclonal antibody, and a total of 10^4^ events for each sample were acquired.

### Western blotting

Aliquots containing 20 μg of proteins from each lysate cells were subjected to electrophoresis on a pre-cast 4% to 15% polyacrylamide gel (BioRad, Hercules, CA, USA) and then transferred to a polyvinylidene difluoride membrane (Trans-Blot Turbo Midi PVDF, 0.2 μM; Biorad) by the Trans-Blot Turbo transfer system (Biorad). Membranes were probed with an anti-LMP7 antibody (1:800, Enzo Life Sciences) and then incubated with secondary antibody. The same membranes were then stripped and proteins were rehybridized with anti-β-actin antibody (mouse; Sigma–Aldrich). Immune complexes were detected by the ECL chemiluminescence system (Amersham Pharmacia, Little Chalfont, UK), as recommended by the manufacturer. Images were acquired using the ChemiDoc imaging system (UVP, Cambridge, UK) and quantified by Image J 1.34 software. The intensity of bands, corresponding to LMP7 proteins, was normalized to the beta actin signal.

### Statistical analysis

Data were expressed as mean ± standard deviation (SD) or standard error of the mean (SEM). Statistical analysis was performed using paired, unpaired Student t-test, as appropriate. Pearson’s correlation test was used to study the association between two continuous variables. A *P* value <0.05 was considered statistically significant. All analyses were performed using GraphPad Prism 5.0 (GraphPad software, Inc., San Diego, CA, USA).

## Results

### Detection of IFN-alpha signature in renal biopsies of patients affected by lupus nephritis

**Figure 1:**
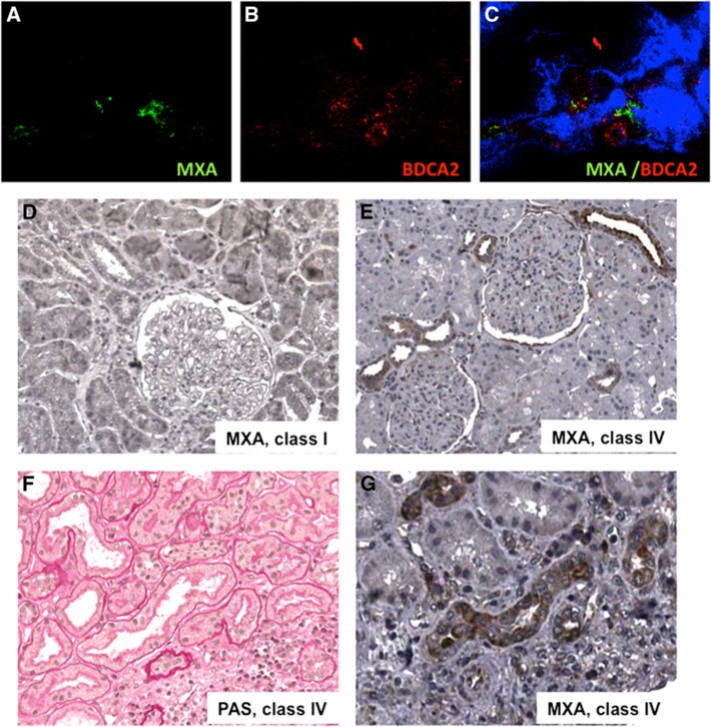
**Detection of IFN-alpha signature in renal biopsies of patients affected by lupus nephritis. (A-C)** Infiltrating plasmacytoid DC in class IV lupus nephritis were characterized with a double staining for MXA (green, **A**) and BDCA-2 (red, **B**), a specific plasmacytoid DC marker (**C**, merge analysis). **(D)** Type I IFN-induced MXA protein was rarely detectable in class I lupus nephritis; **(E)** on the contrary, MXA positive tubular epithelial cells and infiltrating leucocytes were detectable in class IV lupus nephritis. **(F)** PAS staining on a seriate tissue section of the same specimen stained for MXA. **(G)** Immunohistochemistry analysis for MXA was performed on renal biopsies of eight patients for each group, as described in the Methods section. DC, dendritic cells; PAS, Periodic acid-Schiff.

IFN-alpha has a pivotal role in the pathogenesis of autoimmune response in SLE. Therefore, we analyzed renal biopsies from SLE patients affected by lupus nephritis to detect signs of IFN-alpha signaling at the renal level. We used an antibody directed against MXA, a specific protein induced by this type of IFN [17]. As expected (Figure 1A-C), we found that plasmacytoid DC, the major producer of IFN-alpha, stained positive for MXA [17], especially in class IV lupus nephritis, as shown by co-localization of MXA with BDCA2 (Figure 1A-C), a marker of plasmacytoid DCs.

When we analyzed patients with type I/II lupus nephritis, MXA was barely detectable (Figure 1D). On the contrary, we observed a strong expression of MXA at the tubulo-interstitial level in patients with class IV lupus nephritis (Figure 1E). In particular, MXA was detectable in epithelial cells of proximal tubules (Figure 1G), as confirmed by Periodic acid–Schiff (PAS) staining that allows us to distinguish the different structures of the kidney tissue (Figure 1F). MXA was absent in glomeruli, blood vessels and distal tubules (Figure 1E,G).

### Detection of IFN-alpha mRNA in renal biopsies of patients affected by lupus nephritis

**Figure 2:**
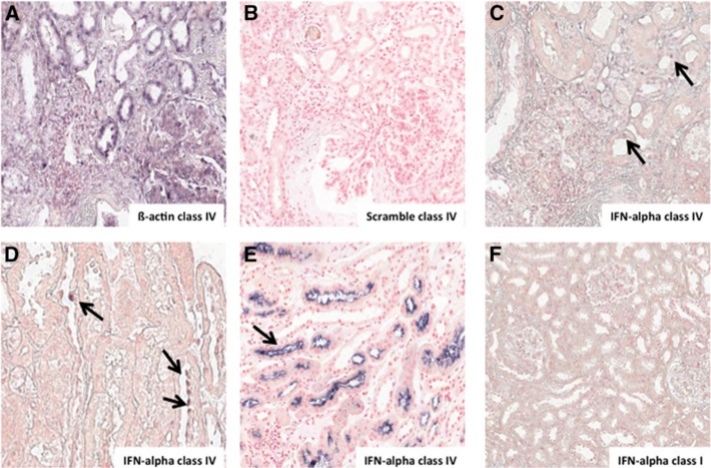
**Identification of IFN-alpha mRNA in renal tubular epithelial cells of patients affected by lupus nephritis. (A)** ß-actin and **(B)** scramble mRNA expression on seriate sections of patients affected by class IV lupus nephritis, representing the positive and negative controls of the ISH reaction, respectively. **(C,D,E)**. ISH mRNA detection on renal biopsies showed that IFN-alpha mRNA was highly expressed in epithelial cells of proximal tubules in patients with class IV lupus nephritis but it was absent in glomeruli, blood vessels and distal tubules. On the contrary, **(F)** IFN-alpha mRNA was marginally expressed in class I lupus nephritis. ISH analysis was performed on renal biopsies of four patients for each group, as described on Methods section. ISH, *in situ* hydridization.

To investigate the possible local synthesis of IFN-alpha, we performed *in situ* hybridization to detect specific mRNA expression (Figure 2). We found that IFN-alpha mRNA was particularly expressed in tubular epithelial cells at the intracellular level (Figure 2C,D; arrows) but also on the luminal side of the tubules in the brush border (Figure 2E; arrows) in patients with class IV lupus nephritis. On the contrary, IFN-alpha mRNA expression was absent in class I/II (Figure 2F).

### Analysis of gene expression profile of IFN-alpha-activated RPTEC

Considering the local production of IFN-alpha by RPTEC associated with the tubular type I IFN signature, we hypothesized a possible autocrine effect of this type of IFN on RPTEC. Therefore, we stimulated RPTEC with IFN-alpha *in vitro* and compared their whole-genome expression profiles with control cells. Following stimulation, we observed 108 significantly up-regulated genes and only 7 down-regulated genes with a fold-change >2 (FDR <0.05). Among them, 12 genes belonged to the gene family coding for IFN-inducible proteins, indicating that RPTEC were capable of responding to IFN-alpha stimulation (Additional file 1). Interestingly, IFN-alpha induced the expression of pivotal genes in the innate immune system, such as complement factor B, which has a critical role in the activation of complement alternative pathway [18], and Toll-like receptor 3 (TLR3) that is primarily involved in the recognition of viral dsRNA [19].

To determine whether there was a coordinated differential expression of a set of functionally related genes in IFN-activated RPTEC, we performed a GSEA, identifying 123 processes differentially regulated by IFN-alpha. As expected, we identified as highly represented (FDR <0.05) the genes involved in the IFN response pathway (Additional file 2). In addition, IFN-alpha induced a coordinated differential expression of gene sets involved in antigen processing and presentation, in response to hypoxia, transplant rejection and, finally, genes associated with SLE pathogenesis (Table 1 and Additional file 2).

**Table 1:** **Most significant processes differentially regulated in IFN-alpha treated RPTEC, identified by GSEA (q values <0.05)**

**Gene set**	**Total genes**	**Genes found**	**q-value [IFN] vs [Control]**	**ES [IFN] vs [Control]**	**NES [IFN] vs [Control]**
KEGG_ANTIGEN_PROCESSING_AND_PRESENTATION	89	88	0.041	0.641	2.293
REACTOME_SIGNALING_IN_IMMUNE_SYSTEM	366	324	0.047	0.415	1.815
PELLICCIOTTA_HDAC_IN_ANTIGEN_PRESENTATION_DN	49	49	0.041	0.683	2.223
LINDSTEDT_DENDRITIC_CELL_MATURATION_A	54	54	0.041	0.665	2.405
ZHANG_RESPONSE_TO_IKK_INHIBITOR_AND_TNF_UP	219	204	0.041	0.575	2.153
SANA_TNF_SIGNALING_UP	75	72	0.041	0.829	2.201
DER_IFN_ALPHA_RESPONSE_UP	57	56	0.041	0.873	2.202
RADAEVA_RESPONSE_TO_IFNA1_UP	32	31	0.047	0.931	1.808
DER_IFN_BETA_RESPONSE_UP	82	79	0.041	0.792	2.474
SANA_RESPONSE_TO_IFNG_UP	68	67	0.049	0.894	1.779
DER_IFN_GAMMA_RESPONSE_UP	58	57	0.041	0.830	2.481
KEGG_ALLOGRAFT_REJECTION	38	38	0.041	0.680	2.254
KEGG_GRAFT_VERSUS_HOST_DISEASE	42	41	0.041	0.656	2.045
FLECHNER_BIOPSY_KIDNEY_TRANSPLANT_REJECTED_VS_OK_UP	88	84	0.041	0.645	2.119
ICHIBA_GRAFT_VERSUS_HOST_DISEASE_D7_UP	133	91	0.041	0.771	2.359
ICHIBA_GRAFT_VERSUS_HOST_DISEASE_35D_UP	158	118	0.041	0.556	2.132
MANALO_HYPOXIA_UP	208	194	0.044	0.473	1.877
MENSE_HYPOXIA_UP	99	87	0.041	0.642	1.897
ELVIDGE_HYPOXIA_UP	174	160	0.041	0.548	2.055
ELVIDGE_HYPOXIA_BY_DMOG_UP	132	122	0.041	0.581	2.017
ELVIDGE_HIF1A_TARGETS_DN	92	83	0.041	0.660	2.011
ELVIDGE_HIF1A_AND_HIF2A_TARGETS_DN	105	95	0.041	0.598	2.068
KEGG_SYSTEMIC_LUPUS_ERYTHEMATOSUS	140	131	0.045	0.547	1.862
BENNETT_SYSTEMIC_LUPUS_ERYTHEMATOSUS	23	23	0.047	0.961	1.802

Among over-expressed genes, we focused our attention on PSMB8, coding for LMP7 protein, a catalytic subunit of the 20S immune-proteasomes (β5i). LMP7 is involved in antigen processing to generate class I binding peptides and acts as a major component of IFN-induced sensitivity. In addition, IFN-alpha induced up-regulation of enzymes involved in protein ubiquitination (DTX3L, FBOX6, UBA7 and UBE2L6), suggesting a significant activation of antigen presentation pathways in RPTECs (Additional file 1) and up-regulation of specific sensors of viral dsRNA (TLR3 and retinoic acid receptor responder 3 (RARRES3); Additional file 1). We then performed a pathway analysis (Ingenuity Pathway Analysis (IPA), Additional file 3) showing that the identified genes, next to the expected link with IFN-alpha signaling, were also strongly connected with the activation of NF-kB pathway.

### Quantitative analysis of IFN-alpha induced genes involved in antigen presentation pathways

**Figure 3:**
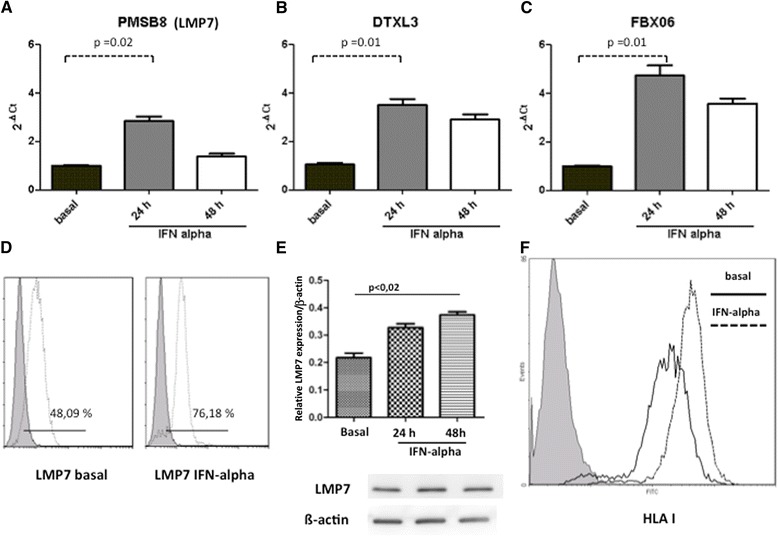
**Quantitative analysis of IFN-alpha up regulated genes and proteins involved in antigen presentation pathways.** Validation of differential expression of PSMB8 **(A)**, DTX3L **(B)** and FBOX6 **(C)** in RPTEC stimulated with IFN-alpha. Expression levels were quantified using RT-PCR. The genes’ relative expressions were normalized to the expression of ß actin. The histograms represent the mean ± SEM. The intracellular expression of LMP7 was evaluated by flow cytometry analysis **(D)** (LMP7 48.09% ± 2 basal versus 76.18% ± 2, 48 hours, 100 U/ml INF-alpha) and western blot analysis (LMP7 basal versus 48 hours, 100 U/ml INF-alpha *P* <0.02) **(E)**. The surface expression of HLA I **(F)** in RPTEC stimulated with IFN-alpha showed a significant increase of mean fluorescence intensity (MnI) (19.5 ± 3 basal versus 43.7 ± 2, 48 hours, 100 U/ml IFN-alpha stimulation). Data shown were gated on RPTEC cells and histograms were based on RPTEC staining with isotype control mAbs. The data presented for both HLA-I and LMP7 are representative of three independent experiments performed using RPTEC cells (*P* <0.02 and *P* <0.05, respectively). mAbs, monoclonal antibodies; RPTEC, renal proximal tubular cells; SEM, standard error of the mean.

We next performed, in a separate set of experiments, RT-PCR to confirm and validate the increased expression of candidate genes identified by microarray analysis. PSMB8, DTX3L and FBOX6 genes showed a significantly increased expression following IFN-alpha stimulation (*P* <0.05; Figure 3A-C). We also quantified intracellular tubular LMP7 by flow cytometer analysis and western blotting. After 48 hours of activation, the synthesis of β5i proteasome subunit LMP7 significantly increased when compared to basal conditions as shown by flow cytometry (Figure 3D) and by western blotting analysis (Figure 3E). In addition, we performed phenotypic analysis of HLA I expression on IFN-activated-RPTEC, confirming the increased surface expression of HLA class I molecules compared to basal conditions (Figure 3F; *P* <0.02).

### Immunoproteasome subunit LMP7 induction in tubular epithelial cells from SLE patients with nephritis

**Figure 4:**
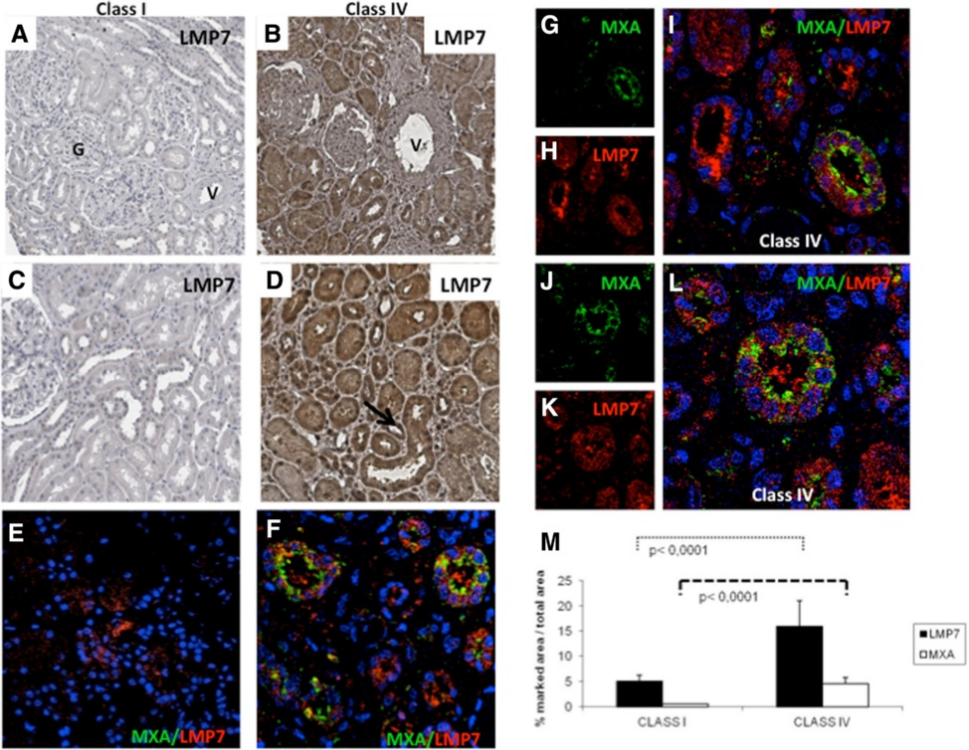
**Immunoproteasome subunit LMP7 induction in tubular epithelial cells from SLE patients with nephritis.** Increased expression of LMP7 was found in renal biopsies of patients with class IV lupus nephritis **(B,D)** compared to those with class I **(A,C)**. LMP7 expression was found at the tubular interstitial level but not on glomerular cells (G) and vessels (V). Co-localization of LMP7 (red **H**, **K**) and MXA (green **G**, **J**) in tubular epithelium of patients with lupus nephritis was investigated by immunofluorescence/confocal microscopy **(E-L)**. We found co-localization of LMP7 and MXA in class IV lupus nephritis **(F,I,L)** but not in class I, where MXA was absent and LMP7 had very low expression **(E)**; nuclei were stained with TO-PRO-3 (blue); **(L)** zoom of figure F. **(M)** Quantification of immunohistochemical staining was carried out as described in Methods section. The histograms represent the increased tissue expressions of MXA (*P* <0.0001) and LMP7 proteins (*P* <0.0001) in eight class IV lupus nephritis patients compared to eight patients of class I/II. SLE, systemic lupus erythematous.

Moreover, we investigated whether the genes and the relative proteins modulated *in vitro* by IFN-alpha were also detectable in patients affected by lupus nephritis. First, we investigated the presence of LMP7 by immunohistochemistry (Figure 4A to D). We observed that LMP7 was expressed at very low levels in tubules of patients with class I/II lupus nephritis, without any expression in glomeruli and blood vessels (Figure 4A and C). On the contrary, we found high LMP7 expression in class IV lupus nephritis, mainly localized at the tubulo-interstitial level (Figure 4B and D). Confocal microscopy analysis showed that the co-localization of MXA with LMP7 was present only in patients with class IV lupus nephritis (Figure 4F,I and L). Quantification of LMP7 and MXA protein expression by immunohistochemistry analysis demonstrated that the difference between the classes was statistically significant (Figure 4M).

### Type I IFN signature is associated with nuclear translocation of pNIK and p65 in tubular epithelial cells

**Figure 5:**
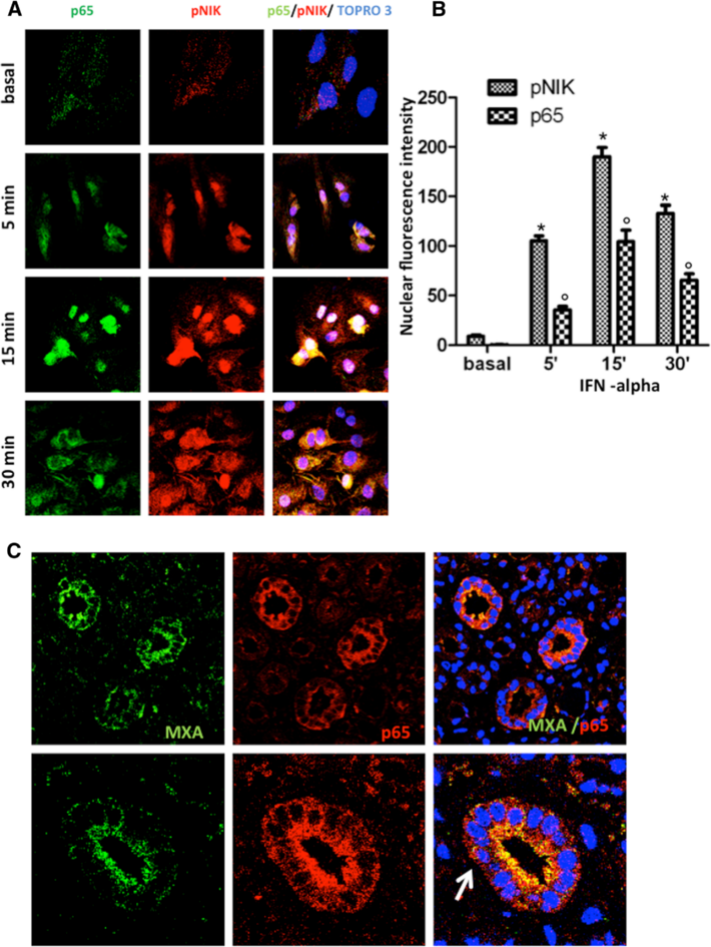
**Activation of NF-kB pathways in tubular epithelial cells with type I IFN signature. (A)** RPTEC were stimulated at different time points with IFN-alpha 100 U/ml and stained for p65 (green) and pNIK (red). At basal conditions both proteins were detectable only in the cytoplasm; after five minutes of stimulation p65 and pNIK began to move from the cytoplasm to the nucleus with significant translocation after 15 and 30 minutes. **(B)** Specific p65 and pNIK fluorescence intensity inside the nuclei was quantified as described in Methods section. Data are averages ± SD for n = 10 cells from one field on one slide; *P <0.05 and °*P* <0.05 for comparison pNIK and p65 versus basal, respectively. The images and results are representative of at least three independent experiments. **(C)** Double staining for MXA (green), p65 (red) and merge analysis (yellow) on class IV lupus nephritis showing translocation of p65 from cytoplasm to nuclei. NF-kB, nuclear factor-kB; RPTEC, renal proximal tubular cells.

As indicated in Additional file 3, the pathway analysis suggested a possible role of IFN-alpha in the activation of the NF-kB pathway in tubular epithelial cells. Therefore, we tested the activation of canonical and non-canonical NF-kB pathways *in vitro* by p65 and pNIK analysis [20]. At basal conditions, p65 was detectable only in the cytoplasm with low pNIK activation (Figure 5A). After five minutes of stimulation with IFN-alpha, p65 and pNIK began to move from the cytoplasm to the nucleus and completely transmigrated in to the nucleus at 15 and 30 minutes (Figure 5A). Interestingly, pNIK presented an intensive migration to the nucleus upon IFN-alpha stimulation [21,22] as indicated by confocal microscopy (Figure 5A). Quantification of specific nuclear fluorescence indicated that nuclear translocation of p65 and pNIK was statistically significant (Figure 5B).

Finally, the analysis of patients affected by class IV lupus nephritis showed that p65 co-localized with MXA (Figure 5C). Interestingly, both MXA and p65 were homogeneously distributed within the cytoplasm, but only p65 was present at the perinuclear and nuclear level, indicating the translocation of p65 from the cytoplasm to the nucleus acting as a transcription factor and activator of inflammatory signaling (Figure 5C).

## Discussion

The major finding of the present study is the demonstration that patients affected by lupus nephritis showed local production of IFN-alpha associated with a type I IFN signature in RPTEC. Therefore, it might be possible to hypothesize that locally produced type I IFN may act with an autocrine effect on RPTEC leading to amplification of the tubulo-interstitial damage in lupus nephritis.

The role of IFN-alpha in the pathogenesis of SLE has been demonstrated both in humans and in various animal models [7,11,23]. However, little is known about the pathogenic mechanisms driven by IFN-alpha leading to the induction and acceleration of lupus nephritis [3]. The pathogenic role of IFN-alpha was first described in pioneer studies on circulating leukocytes from juvenile SLE patients [9,24]. Further studies on micro-dissected glomeruli demonstrated the increased expression of genes directly regulated by type I IFN [25]. However, when we stained renal tissues with an anti-MXA antibody, we found that the effects of type I IFN were mainly detectable within the tubulo-interstitium and not at the glomerular level. These data are in line with our previous observation indicating that plasmacytoid DC, the major producers of IFN-alpha, are mainly recruited at the tubulo-interstitial level [12,13] and rarely detectable within the glomeruli [13]. Plasmacytoid DC are activated by an immune complex of SLE patients via TLR9, resulting in high production of type I IFN [10]. An intra-renal activation of TLR was described and is mediated by an immune complex acting on infiltrating macrophages and dendritic cells [3]. Importantly, only a few drugs, such as hydroxylchloroquine or glucocorticoids in pulse regimen, can interfere with TLR activation in plasmacytoid DC, reducing the production of IFN-alpha [26-29].

TLR activation may also occur on renal resident cells including RPTEC, glomerular endothelium, mesangial cells, and macrophages resulting in the production of large amounts of proinflammatory cytokines, such as type I IFN [3,11]. Interestingly, our data indicated that RPTEC are the major producers of IFN-alpha in lupus nephritis with a particular cellular localization of mRNA transcript.

Effectively, mRNAs might be transported in different subcellular regions for the synthesis of proteins directly to the site of their function. This mechanism allowed the correct distribution of proteins in polarized cells, such as neurons, oocytes, early embryos and epithelial cells. In particular, in polarized epithelial cells the apical or basolateral localization of mRNA reduced cell trafficking and allowed a rapid response to external stimuli [30,31].

The increased recruitment of plasmacytoid DC at the tubule interstitial level [13] and the production of IFN-alpha by RPTEC suggest the presence of specific type I IFN triggers acting at the renal level, a process that might lead to autocrine tubular activation, with production of different mediators of tissue damage such as pro inflammatory cytokines and chemokines [3,32]. Moreover, the induction of MXA in tubular epithelial cells might indicate that these cells are more sensitive to IFN-alpha compared to other resident cells [13].

How type I IFN might be pathogenic for kidney in SLE patients is still debated [3,11]. In a mouse model of lupus nephritis, the pathological lesions and the auto-antibody response were significantly attenuated in mice deficient in type I IFN receptors [33]. Interestingly, we found that IFN-alpha can induce the expressions of innate immune genes in RPTEC, such as complement factor B and TLR3. The local production of complement is also supported by infiltrating leucocytes [32] and might have detrimental effects leading to further activation of tubular epithelial cells expressing C3a and C5a receptors [18,26]. In addition, complement is capable of further increasing the activation of infiltrating B and T lymphocytes driving the local autoimmune response and auto-antibody production [26]. Among the up-regulated genes involved in our analysis, we then focused our attention on genes involved in protein ubiquitination (FBXO6, DTX3L) and in the assembly of immunoproteasome (PSMB8 gene codifying for LMP7 subunit). In particular, we focused our attention on LMP7 (upregulated 3.5 fold), because it belongs to a set of up regulated genes that are part of the same biological process (antigen presentation). In particular, this pathway was highlighted following the application of different statistical approaches (GSEA and the pathway analysis) and represented the biological process most representative in relation to the pathogenesis of the autoimmune diseases. Consequently, LMP7 was identified within the framework of biological processes potentially linked SLE. Most antigenic peptides presented by MHC (Major histocompatibility complex) class I molecules are degraded by intracellular proteins via proteasome. Under inflammatory conditions, the immunoproteasome replaces the standard proteasome, by inducing the new subunits β1i, β2i, and β5i. Once assembled, immunoproteasome significantly contributes to the activation of inflammation via NF-kB [34]. In fact, the LMP7 subunit of immunoproteasome [35] and FBXO6 ubiquitin enzyme, contribute to the increase of degradation of IKBα [20]. In addition, we identified an up-regulation of RARRES 3 that promotes phosphorylation of IKBα, as a first step to the poly-ubiquitination and degradation [36,37]. The pathway analysis indicated a central role of these IFN-regulated genes in NF-kB activation by confirming the co-localization of MXA and p65 in biopsies of patients with class IV lupus nephritis. Particularly interesting is our finding of the nuclear translocation of pNIK upon IFN-alpha activation. pNIK translocation has been involved in nucleosome regulation by enhancing histone H3 phosphorylation [22], a mechanism that might be particularly relevant for autoimmune diseases [1,23]. Moreover, our data contribute to give a new perspective to previous studies showing that RPTEC can participate in immune responses within the kidney and may activate T cells by processing and presenting antigens in an immunogenic form [38].

Our findings indicating that IFN-alpha can induce LMP7 *in vitro* and *in vivo* might also be clinically relevant, since new therapies are currently available that interfere with IFN-alpha signaling, but also with the proteasome/immunoproteasome system [39]. Recent evidence supports the use of proteasome inhibitors in preventing lupus disease progression by targeting both type I IFN activation and auto-antibodies production by plasma cells [40]. It is important to consider that LMP7 can be induced by several cytokines and it might be an interesting target for new therapeutic approaches. By using a specific LMP7 inhibitor *in vivo*, it was possible to suppress arthritis with production of pro-inflammatory cytokines such as TNF-alpha and IL-23 [41], opening new possibilities for the treatment of autoimmune diseases.

## Conclusions

In conclusion, our data indicate that a type I IFN signature is detectable in infiltrating plasmacytoid DC and renal tubular epithelial cells in SLE patients with nephritis, associated with local synthesis of IFN-alpha by RPTEC. By this autocrine loop, type I IFN might have a pathogenic role in lupus nephritis activating pro-inflammatory and antigen presentation pathways in RPTEC. Therefore, inhibition of IFN-alpha signaling might represent a therapeutic strategy to prevent tubulo-interstitial damage in patients with lupus nephritis.

## Additional files

Additional file 1:
**List of genes differentially regulated in IFN-alpha treated RPTEC.** This file contains most significant genes differentially regulated in IFN‐alpha treated RPTEC (FDR <0.05 and fold change >2). Additional file 2:
**Analysis of gene expression profile of IFN-alpha activated RPTEC.** This file contains the significant gene pathways modulated by IFN-alpha. Additional file 3:
**Network analysis.** This file contains the network analysis showing IFN‐alpha modulated genes involved in IFN signaling, NF‐kB pathway and antigen presentation.

## Abbreviations

BDCA2: blood dendritic cell antigen 2
DAB: 3,3′-diaminobenzidine
DC: dendritic cells
DIG: digoxigenin
dsRNA: double-stranded ribonucleic acid
DTX3L: deltex 3 like, E3 ubiquitin ligase
FBS: fetal bovine serum
FBXO6: F-box protein 6
FDR: false discovery rate
GEO: Gene Expression Omnibus
GSEA: gene set enrichment analysis
hEGF: human epidermal growth factor
HLA: human leukocyte antigen
hpf: high power fields
IFN: interferon
IPA: Ingenuity Pathway Analysis
ISH: hybridization *in situ*
LMP7: low–molecular mass polypeptide-7
MnI: mean fluorescence intensity
mRNA: messenger ribonucleic acid
NF-kB: nuclear factor-kB
PAS: periodic acid–Schiff
PBS: phosphate-buffered saline
PSMB8: proteasome subunit, beta type, 8
RARRES3: retinoic acid receptor responder 3
REGM: renal epithelial cell growth medium
RPTECs: renal proximal tubular cells
RT-PCR: real time polymerase chain reaction
SD: standard deviation
SEM: standard error of the mean
SLE: systemic lupus erythematous
TLR3: Toll-like receptor *3*
TLR9: Toll-like receptor 9
UBA7: ubiquitin-like modifier activating enzyme 7
UBE2L6: ubiquitin-conjugating enzyme E2L 6
