# Supplementary material for: Local synthesis of interferon-alpha in lupus nephritis is associated with type I interferons signature and LMP7 induction in renal tubular epithelial cells
Source: Arthritis Res Ther. 2015 Mar 22;17(1):72. doi: 10.1186/s13075-015-0588-3 (PMC4389585; doi:10.1186/s13075-015-0588-3)
Supplement: Additional file 1: — List of genes differentially regulated in IFN-alpha treated RPTEC. This file contains most significant genes differentially regulated in IFN‐alpha treated RPTEC (FDR <0.05 and fold change >2). [file 13075_2015_588_MOESM1_ESM.pdf]

Most significant genes differentially regulated in IFN-alpha treated RPTEC (FDR <0.05 and fold change >2)

Gene Symbol	Definition	Corrected p-value	Fold change	Entrez_Gene_ID
ACO1	Homo sapiens aconitase 1, soluble (ACO1), mRNA.	0.01	-2.3321729	48
APOBEC3G	Homo sapiens apolipoprotein B mRNA editing enzyme, cat	0.08	2.0133746	60489
BATF2	Homo sapiens basic leucine zipper transcription factor, AT	0.02	3.49798	116071
BST2	Homo sapiens bone marrow stromal cell antigen 2 (BST2),	0.01	4.559806	684
BTN3A3	Homo sapiens butyrophilin, subfamily 3, member A3 (BTN	0.08	2.158246	10384
C1orf76	Homo sapiens chromosome 1 open reading frame 76 (C1o	0.05	2.5075312	148753
CAPN13	Homo sapiens calpain 13 (CAPN13), mRNA.	0.04	-2.254069	92291
CASP1	Homo sapiens caspase 1, apoptosis-related cysteine pepti	0.01	2.5312684	834
CD68	Homo sapiens CD68 molecule (CD68), transcript variant 1,	0.04	2.2366548	968
CFB	Homo sapiens complement factor B (CFB), mRNA.	0.02	2.8968248	629
CMPK2	Homo sapiens cytidine monophosphate (UMP-CMP) kinas	0.03	5.8643227	129607
CXCL10	Homo sapiens chemokine (C-X-C motif) ligand 10 (CXCL10)	0.02	3.875868	3627
CYP2J2	Homo sapiens cytochrome P450, family 2, subfamily J, pol	0.06	4.266504	1573
DDX58	Homo sapiens DEAD (Asp-Glu-Ala-Asp) box polypeptide 58	0.06	6.021297	23586
DDX60	Homo sapiens DEAD (Asp-Glu-Ala-Asp) box polypeptide 60	0.05	4.9943247	55601
DHX58	Homo sapiens DEXH (Asp-Glu-X-His) box polypeptide 58 (D	0.01	4.2847238	79132
DTX3L	Homo sapiens deltex 3-like (Drosophila) (DTX3L), mRNA.	0.05	2.0360136	
ECGF1	Homo sapiens endothelial cell growth factor 1 (platelet-de	0.05	3.0638673	1890
EIF2AK2	Homo sapiens eukaryotic translation initiation factor 2-alp	0.05	4.0130863	5610
EIF3EIP	Homo sapiens eukaryotic translation initiation factor 3, su	0.01	-2.1467164	51386
EPSTI1	Homo sapiens epithelial stromal interaction 1 (breast) (EP	0.03	5.2088356	94240
FAM46A	Homo sapiens family with sequence similarity 46, member	0.09	2.3003755	55603
FBXO6	Homo sapiens F-box protein 6 (FBXO6), mRNA.	0.04	2.0799124	26270
FLJ11286	Homo sapiens hypothetical protein FLJ11286 (FLJ11286), r	0.06	2.67841	55337
GBP1	Homo sapiens guanylate binding protein 1, interferon-indu	0.01	3.7977638	2633
GBP4	Homo sapiens guanylate binding protein 4 (GBP4), mRNA.	0.02	5.757413	115361
GMPR	Homo sapiens guanosine monophosphate reductase (GMF	0.04	2.684235	2766
HCG4	Homo sapiens HLA complex group 4 (HCG4) on chromosor	0.02	2.41501	
HCP5	Homo sapiens HLA complex P5 (HCP5), mRNA.	0.02	2.3362038	10866
HERC5	Homo sapiens hect domain and RLD 5 (HERC5), mRNA.	0.02	16.581408	51191

HERC6	Homo sapiens hect domain and RLD 6 (HERC6), mRNA.	0.04	7.112951	55008
HIST1H1C	Homo sapiens histone cluster 1, H1c (HIST1H1C), mRNA.	0.05	2.2291682	3006
HIST1H2BD	Homo sapiens histone cluster 1, H2bd (HIST1H2BD), transc	0.01	2.532493	3017
HIST2H2AA3	Homo sapiens histone cluster 2, H2aa3 (HIST2H2AA3), mR	0.07	4.05535	8337
HIST2H2BE	Homo sapiens histone cluster 2, H2be (HIST2H2BE), mRNA	0.01	2.4645464	8349
HLA-B	Homo sapiens major histocompatibility complex, class I, B	0.03	2.3810048	3106
HLA-C	Homo sapiens major histocompatibility complex, class I, C	0.01	2.311495	3107
HLA-F	Homo sapiens major histocompatibility complex, class I, F	0.01	2.4793148	3134
HLA-H	Homo sapiens major histocompatibility complex, class I, H	0.05	2.1102579	
HNRPA1L-2	Homo sapiens heterogeneous nuclear ribonucleoprotein A	0.02	-3.0194268	
HSD17B14	Homo sapiens hydroxysteroid (17-beta) dehydrogenase 14	0.02	-2.3367577	51171
HSH2D	Homo sapiens hematopoietic SH2 domain containing (HSH	0.01	3.9426036	84941
IFI16	Homo sapiens interferon, gamma-inducible protein 16 (IFI	0.04	3.642999	3428
IFI27	Homo sapiens interferon, alpha-inducible protein 27 (IFI27	0.02	62.201504	3429
IFI35	Homo sapiens interferon-induced protein 35 (IFI35), mRN	0.03	6.1919856	3430
IFI44	Homo sapiens interferon-induced protein 44 (IFI44), mRN	0.02	12.075351	10561
IFI44L	Homo sapiens interferon-induced protein 44-like (IFI44L),	0.06	23.95055	10964
IFI6	Homo sapiens interferon, alpha-inducible protein 6 (IFI6),	0.01	8.648038	2537
IFIH1	Homo sapiens interferon induced with helicase C domain 1	0.06	8.043287	64135
IFIT1	Homo sapiens interferon-induced protein with tetratricope	0.02	18.463058	3434
IFIT2	Homo sapiens interferon-induced protein with tetratricope	0.02	18.433912	3433
IFIT3	Homo sapiens interferon-induced protein with tetratricope	0.04	19.89734	3437
IFIT5	Homo sapiens interferon-induced protein with tetratricope	0.02	2.258807	24138
IFITM1	Homo sapiens interferon induced transmembrane protein	0.02	3.6473453	8519
IL18BP	Homo sapiens interleukin 18 binding protein (IL18BP), tran	0.03	2.9158576	10068
IL4I1	Homo sapiens interleukin 4 induced 1 (IL4I1), transcript va	0.02	2.0285108	259307
IRF7	Homo sapiens interferon regulatory factor 7 (IRF7), transc	0.02	9.219226	3665
IRF9	Homo sapiens interferon regulatory factor 9 (IRF9), mRNA	0.01	2.0619702	10379
ISG15	Homo sapiens ISG15 ubiquitin-like modifier (ISG15), mRNA	0.01	24.423573	9636
ISG20	Homo sapiens interferon stimulated exonuclease gene 20k	0.01	8.739251	3669
KIAA1618	PREDICTED: Homo sapiens KIAA1618 (KIAA1618), mRNA./	0.01	2.9502492	57714
LAMP3	Homo sapiens lysosomal-associated membrane protein 3 (	0.02	8.902856	27074
LAP3	Homo sapiens leucine aminopeptidase 3 (LAP3), mRNA.	0.06	3.2272038	51056

LBA1	Homo sapiens lupus brain antigen 1 (LBA1), mRNA.	0.05	3.1407173	9881
LGALS3BP	Homo sapiens lectin, galactoside-binding, soluble, 3 bindin	0.03	2.6702526	3959
LOC388275	PREDICTED: Homo sapiens similar to Heterogeneous nucle	0.05	-2.7258277	388275
LY6E	Homo sapiens lymphocyte antigen 6 complex, locus E (LY6	0.02	2.9797866	4061
MAFA	Homo sapiens v-maf musculoaponeurotic fibrosarcoma or	0.01	8.795162	389692
MDK	Homo sapiens midkine (neurite growth-promoting factor 2	0.01	2.3128374	4192
NELL1	Homo sapiens NEL-like 1 (chicken) (NELL1), mRNA.	0.07	2.5759199	4745
NMI	Homo sapiens N-myc (and STAT) interactor (NMI), mRNA.	0.02	2.617057	9111
NT5C3	Homo sapiens 5'-nucleotidase, cytosolic III (NT5C3), transc	0.01	4.034263	51251
NUB1	Homo sapiens negative regulator of ubiquitin-like proteins	0.01	2.0760674	51667
OAS1	Homo sapiens 2',5'-oligoadenylate synthetase 1, 40/46kDa	0.05	16.969479	4938
OAS2	Homo sapiens 2'-5'-oligoadenylate synthetase 2, 69/71kDa	0.03	22.336021	4939
OAS3	Homo sapiens 2'-5'-oligoadenylate synthetase 3, 100kDa (	0.04	16.677406	4940
OASL	Homo sapiens 2'-5'-oligoadenylate synthetase-like (OASL),	0.03	9.280907	8638
PARP10	PREDICTED: Homo sapiens poly (ADP-ribose) polymerase f	0.01	2.7967944	84875
PARP12	Homo sapiens poly (ADP-ribose) polymerase family, memb	0.01	4.614056	64761
PARP14	Homo sapiens poly (ADP-ribose) polymerase family, memb	0.03	3.5732002	54625
PARP9	Homo sapiens poly (ADP-ribose) polymerase family, memb	0.01	4.303207	83666
PFKFB4	Homo sapiens 6-phosphofructo-2-kinase/fructose-2,6-biph	0.02	2.3844497	5210
PHF11	Homo sapiens PHD finger protein 11 (PHF11), transcript va	0.05	2.2227407	51131
PLSCR1	Homo sapiens phospholipid scramblase 1 (PLSCR1), mRNA	0.01	2.8826363	5359
PRIC285	Homo sapiens peroxisomal proliferator-activated receptor	0.03	7.599421	85441
PSMB8	Homo sapiens proteasome (prosome, macropain) subunit,	0.05	2.0129988	5696
PSMB9	Homo sapiens proteasome (prosome, macropain) subunit,	0.04	3.5604603	5698
RARRES3	Homo sapiens retinoic acid receptor responder (tazaroten	0.02	2.8509238	5920
RNF213	Homo sapiens ring finger protein 213 (RNF213), mRNA.	0.02	2.3222876	57674
RSAD2	Homo sapiens radical S-adenosyl methionine domain cont	0.01	24.993893	91543
RTP4	Homo sapiens receptor (chemosensory) transporter prote	0.05	5.30767	64108
SAMD9	Homo sapiens sterile alpha motif domain containing 9 (SAI	0.02	5.5380936	54809
SAMD9L	Homo sapiens sterile alpha motif domain containing 9-like	0.05	10.208196	219285
SLC15A3	Homo sapiens solute carrier family 15, member 3 (SLC15A	0.02	8.238367	51296
SLC25A28	Homo sapiens solute carrier family 25, member 28 (SLC25	0.04	2.2958155	81894
SP100	Homo sapiens SP100 nuclear antigen (SP100), transcript va	0.01	2.4937646	6672

SP110	Homo sapiens SP110 nuclear body protein (SP110), transc	0.02	3.6902864	3431
STAT1	Homo sapiens signal transducer and activator of transcript	0.06	2.986846	6772
STAT2	Homo sapiens signal transducer and activator of transcript	0.04	2.5050983	6773
TAP1	Homo sapiens transporter 1, ATP-binding cassette, sub-far	0.05	3.094386	6890
TAP2	Homo sapiens transporter 2, ATP-binding cassette, sub-far	0.01	2.447356	6891
TDRD7	Homo sapiens tudor domain containing 7 (TDRD7), mRNA.	0.08	2.54317	23424
TLR3	Homo sapiens toll-like receptor 3 (TLR3), mRNA.	0.00	2.1839063	7098
TMEM140	Homo sapiens transmembrane protein 140 (TMEM140), m	0.01	2.3016815	55281
TNFSF13B	Homo sapiens tumor necrosis factor (ligand) superfamily,	0.10	5.6416235	10673
TRIM21	Homo sapiens tripartite motif-containing 21 (TRIM21), mR	0.05	2.9740746	6737
TRIM22	Homo sapiens tripartite motif-containing 22 (TRIM22), mR	0.06	3.9143882	10346
TRIM25	Homo sapiens tripartite motif-containing 25 (TRIM25), mR	0.06	2.1047785	7706
TRIM5	Homo sapiens tripartite motif-containing 5 (TRIM5), transc	0.04	2.2433896	85363
TXNDC12	Homo sapiens thioredoxin domain containing 12 (endopla	0.02	-2.378432	51060
UBA7	Homo sapiens ubiquitin-like modifier activating enzyme 7	0.04	4.0850563	7318
UBE2L6	Homo sapiens ubiquitin-conjugating enzyme E2L 6 (UBE2L	0.06	3.3341637	9246
UNC93B1	Homo sapiens unc-93 homolog B1 (C. elegans) (UNC93B1),	0.05	2.3873265	81622
XAF1	Homo sapiens XIAP associated factor 1 (XAF1), transcript v	0.02	14.38809	54739
ZNFX1	Homo sapiens zinc finger, NFX1-type containing 1 (ZNFX1),	0.06	2.5432763	57169
