# Supplementary material for: Local synthesis of interferon-alpha in lupus nephritis is associated with type I interferons signature and LMP7 induction in renal tubular epithelial cells
Source: Arthritis Res Ther. 2015 Mar 22;17(1):72. doi: 10.1186/s13075-015-0588-3 (PMC4389585; doi:10.1186/s13075-015-0588-3)
Supplement: Additional file 2: — Analysis of gene expression profile of IFN-alpha activated RPTEC. This file contains the significant gene pathways modulated by IFN-alpha. [file 13075_2015_588_MOESM2_ESM.pdf]

Additional file 2

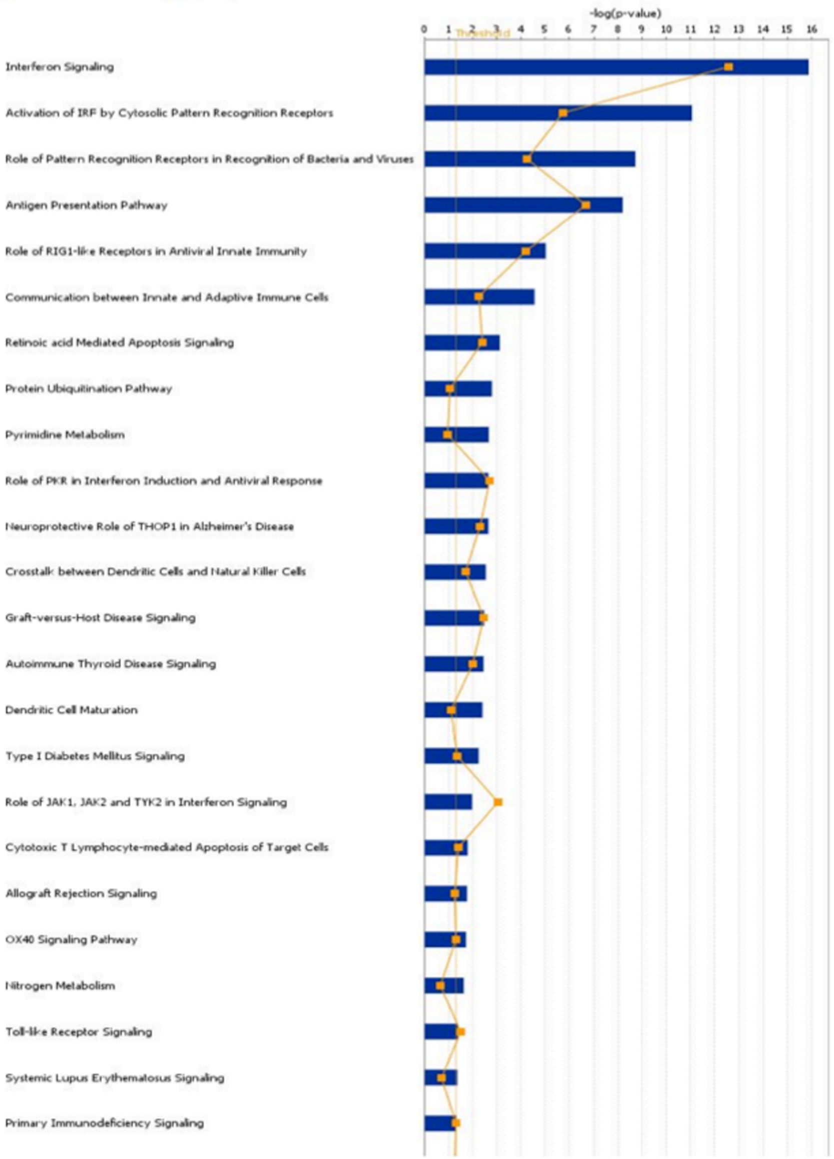

Analysis of gene expression profile of IFN-alpha activated RPTEC. Bioinformatics analysis of significant pathways in which genes modulated by IFN-alpha are involved.
