# Supplementary material for: Local synthesis of interferon-alpha in lupus nephritis is associated with type I interferons signature and LMP7 induction in renal tubular epithelial cells
Source: Arthritis Res Ther. 2015 Mar 22;17(1):72. doi: 10.1186/s13075-015-0588-3 (PMC4389585; doi:10.1186/s13075-015-0588-3)
Supplement: Additional file 3: — Network analysis. This file contains the network analysis showing IFN‐alpha modulated genes involved in IFN signaling, NF‐kB pathway and antigen presentation. [file 13075_2015_588_MOESM3_ESM.pdf]

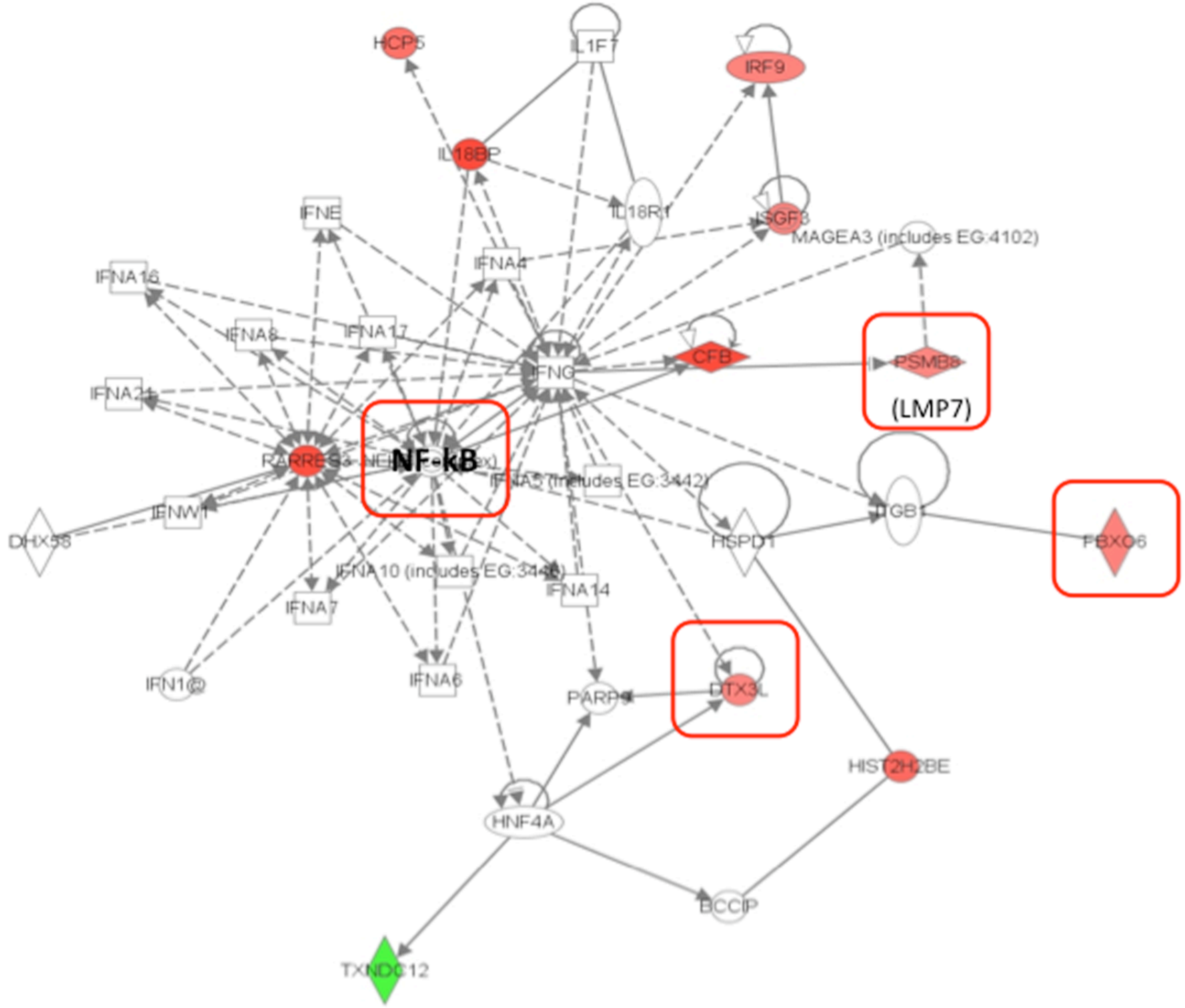

Network analysis showing IFN-alpha modulated genes involved in IFN signaling, NF-kB pathway and antigen presentation. The analysis of this top-ranked network revealed an important role for immunoproteasome subunit LMP7 and ubiquitins (FBXO6 and DTX3L genes). Significant is also the activation of NF-kB pathway by means of RARRES3 and IFN regulatory factors. The network is graphically represented as nodes (genes) and edges (the biological relationship between genes). Red and green shaded nodes represent upregulated and down regulated genes, respectively.
